# Study on SO_4_^2−^/Cl^−^ Erosion Resistance and Mechanism of Recycled Concrete Containing Municipal Solid Waste Incineration (MSWI) Powder

**DOI:** 10.3390/ma15155352

**Published:** 2022-08-03

**Authors:** Yun Dong, Yuanshan Ma, Ningbo Peng, Jianchun Qiu

**Affiliations:** 1 Faculty of Architecture and Civil Engineering, Huaiyin Institute of Technology, Huai’an 223001, China; dyun@hyit.edu.cn (Y.D.); pengnb@hyit.edu.cn (N.P.); 2 College of Water Conservancy and Hydropower Engineering, Hohai University, Xikang Road No.1, Nanjing 210098, China; 3 College of Hydraulic Science and Engineering, Yangzhou University, Yangzhou 225009, China; qiujc@yzu.edu.cn

**Keywords:** municipal solid waste incineration (MSWI) powder, strength activity, salt solution erosion, capillary migration parameters

## Abstract

In this paper, the strength characteristics and erosion resistance of solid waste incineration (MSWI) powder were studied. Firstly, the optimum process for the preparation of regenerated powder from MSWI bottom slag by ball milling was determined as follows: rotational speed 350 r/min, time 45 min. The strength activity index of regenerated powder reached the maximum when the substitute content of powder was 30%. Secondly, the semi-erosion method was used to study the strength variation rule of mortar with different content of MSWI powder in semi-immersion of salt solution. It was found that the higher the content of MSWI powder, the greater the anti-erosion coefficient of mortar specimen. Finally, the capillary rise test, crystallization test and capillary pore water absorption test were used to study the total porosity, coarse capillary-pore porosity and fine-capillary pore porosity of concrete containing MSWI powder. The results showed that, with the increase in MSWI powder content, the above pore structure properties were improved. The results revealed the transport and crystallization process of salt solution in concrete mixed with MSWI powder and the mechanism of corrosion resistance.

## 1. Introduction

With the continuous development of construction engineering, concrete structure is widely used in construction, bridge, road and other structural engineering. The durability mechanism of concrete structure has become the focus of global attention, which is the most critical index affecting construction quality. In practical engineering, the durability of concrete materials is mostly damaged by sulfate and chloride, which is also the most complicated and destructive cause of concrete structure damage [1,2,3,4,5].

Sodium sulfate was the most widely distributed sulfate solution in nature and had the most serious erosion to concrete [6,7]. According to experimental studies [8,9,10,11,12,13,14,15], the crystallization of sodium sulfate consists of three compounds, which were Na_2_SO_4_-Thenardite, Na_2_SO_4_·10H_2_O-Mirabilite, and Na_2_SO_4_·7H_2_O-Sodium sulfite heptahydrate. Among them, Na_2_SO_4_·10H_2_O was difficult to maintain at a stable state and was easily decomposed into Na_2_SO_4_ and Na_2_SO_4_·7H_2_O. The changes of temperature and humidity also had a significant influence on the chemical composition of sodium sulfate crystals. The sulfate resistance of concrete was affected by its shape and appearance, permeability resistance and compactness [16,17,18,19,20]. The corrosion of concrete by chloride salt was mainly reflected in the corrosion of steel bars inside concrete. The transmission mechanism of sodium chloride in concrete was complex. Generally, chloride ions invade into concrete through three different combinations of diffusion, penetration and adsorption capillary [21,22,23,24]. The main factors of concrete’s resistance to chloride ion erosion were the porosity and pore structure of concrete and the amount of chloride-ion binding of the concrete [25,26,27,28]. The smaller the porosity and pore size of concrete, the worse the ability of chloride ions to enter concrete. The concrete’s stronger ability to bind chloride ions slowed down the penetration rate of chloride ions. Under capillarity and diffusion conditions, chloride ions eroded cement-based materials at different rates and paths, which were related to structural pore characteristics and the combination of chloride ion and cement-based materials.

In view of the study on the durability of concrete by mineral admixtures, Qin Lixiang believed that using more fly ash instead of cement can reduce the pore structure of cement concrete and the content of Ca(OH)_2_ so as to improve the structure of concrete transition zone and enhance the sulfate erosion resistance of concrete. Fly ash can also increase the resistance of HPC to sulfate corrosion [29]. When the slag content in concrete reached 70%, it can resist the erosion of most sodium sulfate crystals. Wang Yanmou et al. found that the anti-erosion performance of concrete needed a slag content not less than 65% [30], but the curing conditions of slag Portland cement were harsh. The curing temperature also harmed the sulfate corrosion resistance of cement concrete [31]. The addition of silica powder in concrete can significantly improve its sulfate resistance. Yue Xibing et al. indicated that the addition of slag and silica powder did make concrete more compact [32], and the reduction in cement content also reduced the Ca(OH)_2_ generated in the secondary hydration reaction of concrete; thus, the sulfate resistance of concrete can be improved. However, as a new type of admixture, there is little research on urban-life incinerations for bottom slag with respect to the durability of concrete.

In this paper, the anti-SO_4_^2−^/Cl^−^ solution erosion performance of mortar samples containing MSWI was studied to discuss the variation of anti-corrosion coefficient with the amount of fine powder. The crystallization transport process of salt solution in bottom slag recycled concrete was also studied. By studying the change of porosity of recycled concrete containing MSWI bottom slag and the correlation between capillary pore gaps and different factors, the mechanism of the salt crystallization resistance of recycled concrete containing MSWI bottom slag was revealed.

## 2. Materials and Test Methods

### 2.1. Material

**Figure 1:**
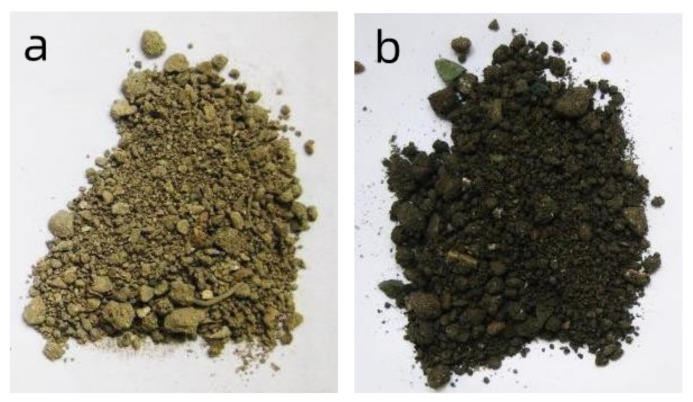
Bottom ash samples after (**a**) and before drying (**b**).

**Figure 2:**
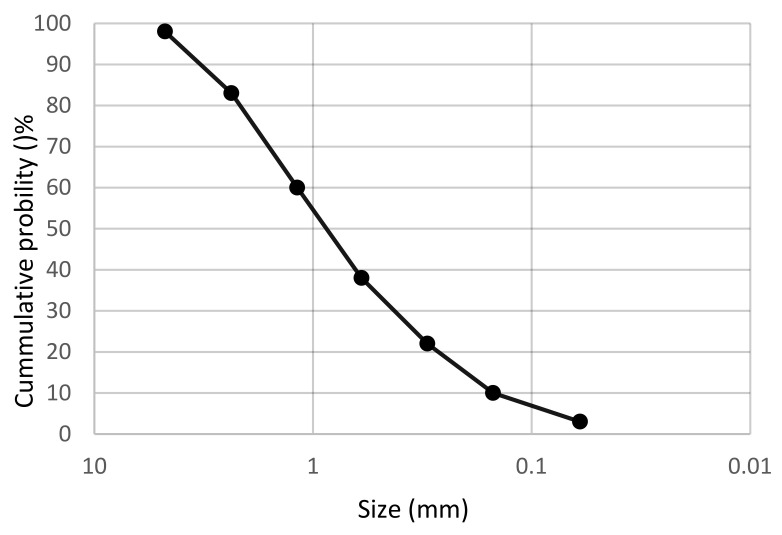
Gradation curve of MSWI powder.

The MSWI residue used in this experiment was obtained from a waste incineration power plant in Huai’an city, Jiangsu Province. The samples randomly selected on site were generally gray and black, and after drying, they were gray and white with a slight odor, as shown in Figure 1. Block particles, incomplete combustion compounds, iron and other materials in the waste incineration bottom slag were screened out and removed. Impurities in the waste incineration bottom slag were washed and removed to ensure the activity of the bottom slag and the strength of mixed concrete. The particle size distribution curve of the bottom slag is shown in Figure 2. The results indicated that the non-uniformity coefficient was 1.7 and the curvature coefficient was 1.3, and the particle size distribution of bottom slag was relatively uniform [33]. The chemical composition of MSWI residue was tested by Philips PW2400 X-ray fluorescence spectrometer. The chemical composition results of MSWI residue are shown in Table 1.

The ordinary Portland cement of strength grade 42.5 was used, which was in line with the general Portland cement (GB175-2009) standard provisions. The adoption of standard sand was in line with China’s new standard ISO. The industrial grade anhydrous sodium sulfate and anhydrous sodium chloride were >99.0% and 99.5%, respectively. The recycled concrete containing MSWI was tested using natural sand and gravel in accordance with JCJ 52-2006 standards, and polycarboxylic acid high-efficiency water-reducing agent was used as the water-reducing agent.

### 2.2. Test Method

#### 2.2.1. Determination of Pretreatment Process

In this experiment, the influence of ball mill rotation speeds and ball grinding time on MSWI residue regeneration was considered. Firstly, the grinding time was 30 min, and the bottom slag was ground at the rotation speed of 200 r/min, 250 r/min, 300 r/min, 350 r/min, 400 r/min, and 450 r/min to obtain the fine powder. The residue ratio was measured by a 80-mesh sieve, and the specific surface area of MSWI residue was measured by a laser particle size analyzer to find out the optimal speed of the ball mill under fixed grinding time. Under the optimum speed of ball mill, the influence of time factors on the regeneration efficiency of MSWI bottom slag was determined. The bottom slag was ground for 15 min, 30 min, 45 min and 60 min at the optimal speed, and the screening residual rate of micro-powder was determined using a 80-mesh sieve. The specific surface area of micro-powders was determined by a laser particle-size analyzer, and the optimal ball milling time was determined.

#### 2.2.2. Strength Test of MSWI Slag Regenerated Micro-Powder

The Dkz-6000 electric bending test machine and automatic constant pressure compression test machine are used for mortar strength tests. According to the method required in GB17671-1999 [34], the mortar molding size is 40 mm × 40 mm × 160 mm, and the casting is carried out by a combined triplet test mold. Under the premise of maintaining the same water-binder ratio (W/C = 0.5), the same amount of cement was replaced by the regenerated micro-powder from the bottom slag. Experiments were carried out with different proportion of micro-powder content in nine groups. The mass fraction of regenerated micro-powder replacing cement was 10%, 12.5%, 15%, 17.5%, 20%, 22.5%, 25%, 27.5%, and 30%, respectively, as shown in Table 2.

#### 2.2.3. Erosion Test of Mortar Containing MSWI

**Figure 3:**
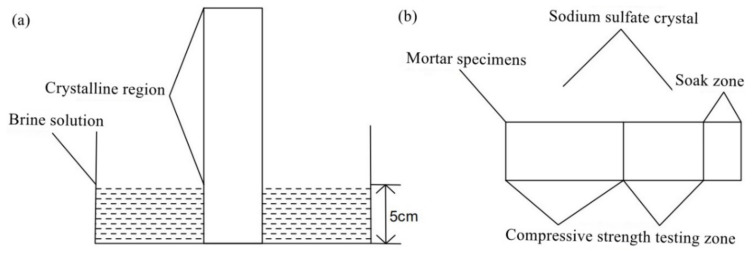
Diagrams of semi-immersion test (**a**) and compressive strength test (**b**).

The erosion and damage of sodium sulfate crystals on mortar specimens containing MSWI were investigated in the laboratory. Sodium sulfate solution mainly entered cement-based materials through capillary action. In this experiment, the method of semi-immersion experiment was adopted, and the mortar specimen was formed by a triple mold of 40 mm × 40 mm × 160 mm. After standard curing for 28 days, the specimens were divided into two groups, which were first heated and dried in the oven. The first group was half-soaked in clean water, and the second group was half-soaked in 5% sodium sulfate solution (see Figure 3). The effect of sodium sulfate solution on the compressive strength and flexural strength of the unsoaked part (crystalline area) of MSWI residue samples was studied. The schematic diagram of the semi-immersion experiment is shown in Figure 3a. The compressive and flexural test area is shown in Figure 3b. In the test area, sodium sulfate was formed by an aggregation of sodium sulfate crystals on the surface of the mortar under the action of capillary rise.

After the mortar specimen containing MSWI was corroded by sodium sulfate solution, the specimen expanded and its surface spalled, resulting in mass loss. The water on the surface of the specimen dried and then the water was poured on an electronic scale for quality measurements. The accuracy of electronic scale needed to reach 0.01 so as to accurately calculate the rate of mass change. The mass change rate of specimens is calculated by Formula (1):

(1) $$w_{t}=\frac{m_{t}-m_{0}}{m_{t}}\times 100\%$$

where *w_t_* is the mass change rate of the specimen when the age of erosion is *t*; *m_t_* is the mass of the specimen when the erosion age is *t*, unit/kg; *m*_0_ is the initial mass of the specimen, unit/kg.

The influence of physical erosion of mortar specimen containing MSWI by sodium sulfate crystallization can be evaluated by calculating the corrosion resistance coefficient. The compressive and corrosion resistance coefficient referred to the ratio of the compressive strength of specimens with the same curing age and the same mixing ratio soaked in salt solution and water. The flexural and corrosion resistance coefficients referred to the ratio of flexural strength of specimens with the same curing age and the same mix soaked in salt solution and clean water, as described in Formulas (2) and (3).

(2) $$K_{mi}=\frac{R_{mi}}{R_{mi}}\times 100\%$$

(3) $$K_{ni}=\frac{R_{ni}}{R_{ni}}\times 100\%$$

*K_mi_* is the compressive corrosion resistance coefficient of the specimen at the *i* th erosion age; *R_mi_*_1_ is the compressive strength of the specimen at the *i* th erosion age in salt solution, unit/MPa; *R_mi_*_2_ is the ith erosion age of the specimen in clear water compressive strength, unit/MPa; *K_ni_* is the flexural and corrosion resistance coefficient of the specimen at the *i* th erosion age; *R_ni_*_1_ is the flexural and corrosion-resistant coefficient of the specimen in the salt solution at the *i* th erosion age, unit/MPa; *R_ni_*_2_ is the compressive strength of the specimen at the *i* th erosion age in clean water, unit/MPa.

For chloride ion erosion tests of mortar containing MSWI, S1–S4 samples with different dosage of MSWI were prepared according to Table 3. After curing for 28 days, the surface layer was removed and then mashed. A sieve of 1.20 mm and 0.2 mm was used to collect particles with particle size between 1.20 mm and 0.2 mm. The specimen was first placed in an oven and dried at 60 °C for 6 h to remove water in cement-based materials reclaimed from MSWI slag, thus stopping the hydration reaction [35]. The 35 g of pounded MSWI was weighed, and 100 mL of sodium chloride solution with chloride ion concentration of *C*_0_ was taken from the mixture of sodium chloride and saturated Ca(OH)_2_. Cement-based materials and sodium chloride solution were sealed in a beaker and then placed in a curing chamber for one week.

After a week, the transparent liquid was removed from the beaker and its chloride ion concentration *C*_1_ (mol/L) was measured. Then, continue to add 50 mL (*V*_4_) of distilled water and a small amount of Ca(OH)_2_ solution into the beaker to make the solution a saturated Ca(OH)_2_ solution. The beaker was sealed in the curing chamber for three days, and the equilibrium solution was taken out from the beaker. The concentration of chloride ions in the equilibrium solution was *C*_2_ (mol/L), and the formula is shown in (4):

(4) $$C_{1}=\frac{C_{0}V_{1}}{20};C_{2}=\frac{C_{0}V_{2}}{20}$$

where *C*_01_ is the concentration of AgNO_3_, unit mol/L; *V*_1_ is the capacity of AgNO_3_ consumed by the first titration, unit mL; *V*_2_ is the capacity of AgNO_3_ consumed by the second titration, unit mL.

The calculation formulas for the binding amount of chloride ions are shown in (5), (6), (7) and (8):

(5) $$W_{1}=\frac{35.45V_{0}(C_{0}-C_{1})}{M_{g}}$$

(6) $$M_{g}=\frac{(1+m_{0})W_{c}\alpha }{1+m_{0}W_{c}\alpha }m$$

(7) $$W_{2}=\frac{\left[C_{0}V_{0}-C_{1}V_{3}-C_{2}\left(V_{0}+V_{4}-V_{3}\right)\right]}{M_{g}}$$

(8) $$W_{3}=W_{2}-W_{1}$$

where *V*_0_ volume is 100 mL; *C*_0_ is the concentration of NaCl solution saturated with Ca(OH)_2_; *W*_1_ is the binding amount of all chloride ions (physical adsorption and chemical binding), in unit 10^−3^; *M_g_* is the total amount of gel material (cement and micro-powder), unit g; m is dry sample mass, unit g; *m*_0_ is the water requirement of cementing material (cement and powder) per unit mass, and the value is 0.25; *W*_1_ is the dimensionless ratio of cementitious material to the total amount of cementitious material and sand; *α* is the degree of hydration, *α* = *M*_1_/*m*_0_; *W*_2_ is the chemical binding amount of chlorine ion per unit mass of gel (cement and micro-powder), unit 10^−3^. *W*_3_ is the physical adsorption amount of chloride ion per unit mass of gel (cement and micro-powder), unit 10^−3^; *V*_3_ is the first equilibrium solution used for titration, unit mL.

#### 2.2.4. Strength of Recycled Concrete Containing MSWI

All concrete specimens used in this experiment were tested in accordance with the standard JGJ 55-2000 [36], and the concrete size was 150 mm × 150 mm × 150 mm and 100 mm × 100 mm × 100 mm. In the experiment, the recycled concrete adopts a 0.4 water-binder ratio. According to the premise of keeping the water-binder ratio unchanged, the substitute cement is mixed with micro-powder, and four groups of parallel experiments with different replacement ratios are carried out. The mass ratios of recycled micro-powder replacing cement were 0%, 10%, 20% and 30%, respectively, as shown in Table 4. The compressive strength of the MSWI bottom slag recycled-concrete test block was tested by a YAD-2000 pressure testing machine. In the experiment, the recycled concrete adopted 0.4 w/c ratio, and on the premise of keeping the w/c ratio unchanged, the micro-powder was replaced by cement for 4 different parallel experiments. The mass ratios of recycled micro-powder replacing cement were 0%, 10%, 20% and 30%, as shown in Table 4. The compressive strength of MSWI bottom slag recycled concrete test block was tested by a YAD-2000 pressure testing machine.

The porosity of concrete can be obtained indirectly from the water loss rate of saturated concrete specimen. Concrete samples were made of 100 mm × 100 mm × 100 mm by the vibration molding method. After curing for 28 d and vacuum saturation, the water on the surface of the concrete samples was wiped with a dry cloth first. The mass of the samples was *m*_1_ measured by an electronic balance. Then, it was placed in the curing chamber at 90% relative humidity for 30 days. When water diffusions in concrete reache equilibrium state, the mass *m*_2_ of the same specimen was measured again. Under the condition of T = 105 °C, the specimen was dried to a constant weight and finally weighed *m*_3_ after cooling to calculate concrete porosity. The specific equations are shown in (9), (10) and (11):

(9) $$P_{1}=\frac{\left(m_{1}-m_{2}\right)}{m_{1}\rho_{w}}\times 100\%$$

(10) $$P_{2}=\frac{\left(m_{1}-m_{3}\right)}{m_{1}\rho_{w}}\times 100\%$$

(11) $$P_{3}=P_{2}-P_{1}$$

where *P*_1_ is the coarse capillary porosity; *P*_2_ is the total porosity; *P*_3_ is the fine capillary porosity; *M*_1_ is the mass of saturated, unit kg; *M*_2_ is the mass of concrete specimen placed at 90% relative humidity for 30 d, unit kg; *M*_3_ is the mass of dry concrete specimen, unit kg; *ρ*_1_ is the density of concrete specimen, unit kg/m^3^; *ρ_w_* is the density of water in kg/m^3^.

**Figure 4:**
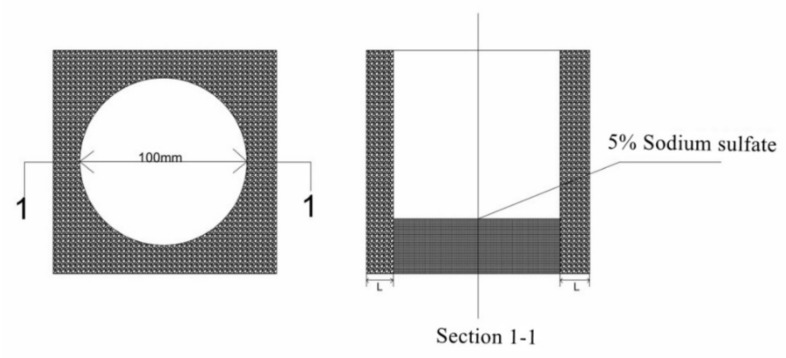
Schematic diagram of crystallization experiment.

For the capillary rise experiment and capillary crystallization experiment of reclaimed concrete containing MSWI, 150 mm of side length concrete was first made according to the mix ratio. After 28 d curing in the curing room, a cylinder with a diameter of 100 mm and height of 150 mm was drilled on the concrete specimen with the core machine, and moisture was dried in the oven. The core body was half immersed in sodium sulfate solution, and the rising height of the surface of the core body of different specimens was measured. The remaining holes of concrete after core removal were closed. In the use of 5% sulfuric acid solution drops into the hole, crystallization from the surface of the concrete test block penetration time T and the concrete side wall thickness L were tested. Through the relation between the time T of crystallization penetrating out from the surface of concrete test block and the inner wall thickness L of concrete, the formula *V*_S_ = L/T of osmotic crystallization rate is calculated, and the unit is cm/s. The experiment was carried out at room temperature, and the experimental device is shown in Figure 4.

**Figure 5:**
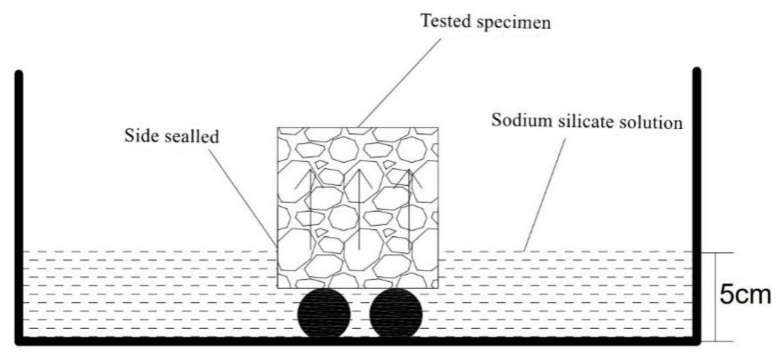
Capillary water absorption experiment of MSWI recycled concrete.

For capillary water absorption tests of reclaimed MSWI concrete, a concrete cube with a side length of 100 mm should be made. After curing for 28 d, it should be sealed with oxidized grease around it and placed into a drying box for drying. The test block was semi-immersed in a solution of sodium sulfate with a water surface height of 5 cm. It was taken out every once in a while and dried with a wet cloth. The weight of the test piece was immediately weighed. The experimental measurement time was the accumulated water absorption Δ*W* at 10 time points in 24 h. The experimental device is shown in Figure 5.

## 3. Results and Discussion

### 3.1. Determination of Pretreatment Process

**Figure 6:**
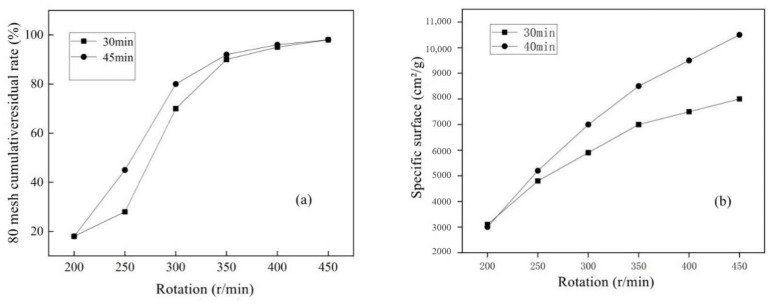
Screen residual rate of 80-mesh (**a**) and specific surface (**b**) versus different rotating speeds of MSWI mortar.

At a fixed time of 30 min and 45 min, the cumulative pass rate of 80 mesh corresponding to different speeds is shown in Figure 6a. Under different grinding times, the change trend of 80-mesh cumulative pass rates of MSWI bottom slag was basically the same. In general, the fineness of fine powder increases with the increase in ball grinding speeds. When the rotational speed was low, the 80-mesh passing rate of the regenerated powder increased with the rotational speed. However, the fineness of MSWI slag regenerated with the ball milling speed of 300~350 r/min increased with the rotation speed. In this experiment, when the grinding times of the ball mill are 30 min and 45 min, the passing rate of MSWI bottom slag regenerated micro-powder 80 mesh increased by 150% and 87% compared with 300 r/min and 250 r/min, respectively. For grinding times of 30 min, the pass rate of 400 r/min increased by 4.8% compared to 350 r/min, and the pass rate of 450 r/min increased by 2.3% compared to 400 r/min. It can be seen that when the rotation speed reached 400 r/min, the 80-mesh passing rate of the regenerated micro-powder had little changes.

The specific surface area of MSWI powder at different rotational speeds is shown in Figure 6b. When the milling time was 30 min, the specific surface area of the regenerated powders increased by 38%, 22.8%, 17.8%, 11.6%, and 10.7% and an increase in rotating speeds was observed. When the milling time was 45 min, the specific surface area increased by 64.3%, 29.3%, 19.4%, 15.2% and 11.4%. The specific surface area of MSWI powders increased gradually with the increase in rotating speed, but the increasing degree of MSWI powders decreased obviously. In the ball milling process, the volume of the bottom slag decreased to a certain extent and became fine powder particles. As the grinding speed increases, the volume continues to decrease mainly by the friction with the steel ball rather than the contact, friction and collision between the bottom slag of large particles. Therefore, late ball milling of fine foundation slag was similar to rolling frictions, and rolling friction was related to the roughness of contact surface. When the fine foundation slag became fine, the contact surface of the steel ball became smoother and the rolling friction decreased, which made the specific surface area of the fine foundation slag rise slowly.

Based on the above experimental results and analysis, when the rotational speed was 350 r/min, the residue ratio of 80 mesh regenerated powder had reached a high level, and the specific surface area of the regenerated powder reached 7000–8000 cm^2^/g, which obviously exceeded the specific surface area specification of conventional cement. The continuous increase in speed accelerates the wear of the machine and the consumption of electric energy; thus, 350 r/min was selected as the optimal speed.

### 3.2. Strength Test Results of Mortar Containing MSWI

The compressive test results and strength activity indexes of different powder dosages are shown in Table 5. With the increase in MSWI powder content, the compressive strength of mortar specimens at different ages gradually decreased. When the content of regenerated powder was 30%, the strength activity of mortar specimen was about 55%. The age of strength difference between high-content and low-content mortar specimens was 3 d > 7 d > 14 d > 28 d. Compared with the early strength, MSWI powder had a significant influence on the late strength of mortar specimen. The MSWI powder had a certain strength activity, and its late strength and early strength growth rate of mortar specimens were large. However, the active strength index of mortar with low content can reach 80% in the later stage, and the strength growth rate in the early stage was fast, so it was important to choose a more appropriate content of fine powder.

The test results of 28 d flexural strength of mortar containing MSWI with different dosage are shown in Table 6. The flexural strength and compressive strength of mortar containing MSWI bottom slag recycled had the same trend. The flexural strength decreased with the increase in MSWI powder. When the replacement rate was 30%, the flexural strength activity index was less than 50%, which was less than the compressive strength activity index.

The content of calcium oxide in MSWI powder was lower than that of cement, and there were few effective hydration products that can promote strength growth in the early stage. Therefore, the strength of mortar specimens containing MSWI was lower than that of pure mortar. On the other hand, MSWI powder was rich in silicon oxide and alumina, which can promote the pozzolanic reaction and effectively increase the later strength of cement-based materials. When the amount of MSWI powder was small, aluminum acid ions, sulfate ions and some unhydrated calcium oxide in cement-based materials were hydrated and regenerated into crystals, thus increasing the strength of mortar. However, the mass substitution of MSWI powder significantly reduced the content of calcium oxide in the paste, resulting in a reduction in crystals formed by aluminate ions as sulfate ions.

### 3.3. Test Results of Cl^−^ and SO_4_^2−^ Erosion Resistance of Mortar Containing MSWI

**Figure 7:**
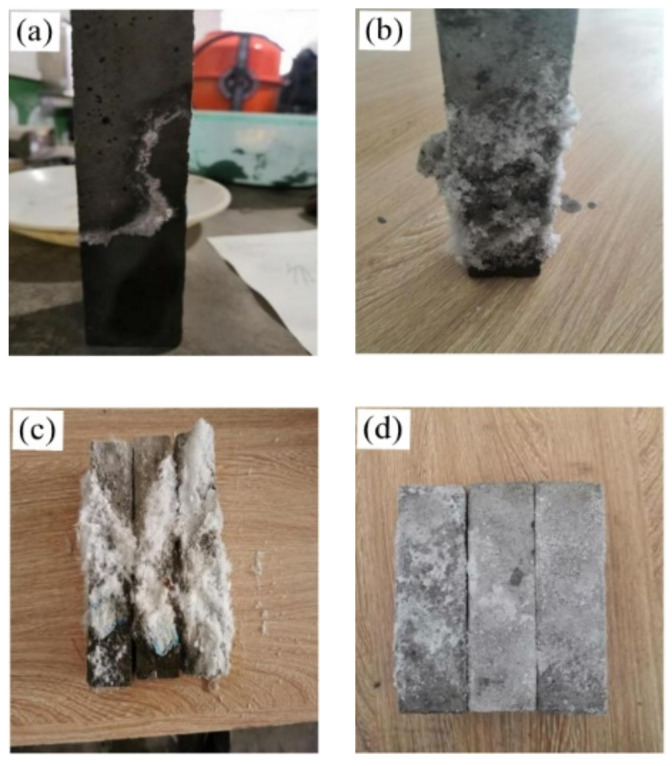
Mortars specimen morphology under semi-immersion erosion. (**a**) one month, (**b**) three months, (**c**) six months, (**d**) removed the crystallization.

Figure 7 shows the mortar appearance changes of semi-immersed mortar specimens in different periods. A similar white crystalline crack permeated the surface of the mortar specimen after half soaking for one month, but the specimen surface remained intact, as shown in Figure 7a. After half soaking for three months, a large number of white crystals appeared on the mortar surface, and the specimen surface remained intact, as shown in Figure 7b. After soaking for six months, the surface of the mortar was covered with white crystals, which were stored in blocks. The crystals outside the block crystals were cotton-like, as shown in Figure 7c. The crystallization was removed from the surface of the mortar, the specimen was characterized by obvious spalling, and some moderate transverse or vertical cracks appeared at the mortar specimen, as shown in Figure 7d.

**Figure 8:**
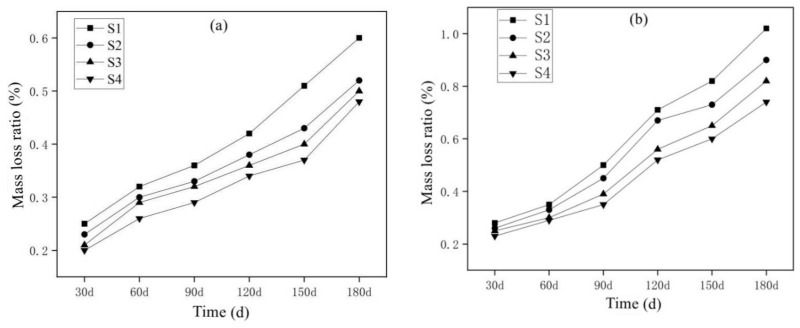
Mass loss rate of specimens soaked in water (**a**) and sodium sulfate solution (**b**).

Figure 8a shows the mass change of the MSWI-containing mortar specimens under long-term semi-immersion in clean water. The mass of the semi-immersed specimens in clear water increased slightly with the increase in erosion age, and the mass of S1~S4 increased, respectively, when immersed for 6 months at 0.6%, 0.52%, 0.5% and 0.48%. Figure 8b is the mass change rate of the MSWI-containing mortar specimen under the condition of long-term sodium sulfate semi-immersion. As erosion age increases, the quality of the specimen also increased. When soaked for 6 months, the mass of S1~S4 increased by 1.02%, 0.90%, 0.82% and 0.74%, respectively. The mass increase rate of the mortar specimen was obviously larger than that of the MSWI-containing mortar specimen under the condition of long-term semi-immersion in clean water. The crystallization of sulfuric acid due to ion exchanges can increase the quality of the test piece. Even if the Ca(OH)_2_ in the mortar specimen dissolves, SO_4_^2−^ radical ions in the external solution also enter the interior of the specimen to carry out chemical reactions, thereby increasing the mass of the MSWI-containing mortar specimen [37]. It can be seen from Figure 8b that the mass increase in the S_1_ specimen was 12% higher than that of the S_2_ specimen when eroded for 180 d. The mass increase in the S_1_ specimen was 20% higher than that of the S_3_ specimen, and the mass increase in the S_1_ specimen was 27% higher than that of the S_4_ specimen.

#### 3.3.1. Results of SO_4_^2−^ Corrosion Resistance of Mortar Containing MSWI

**Figure 9:**
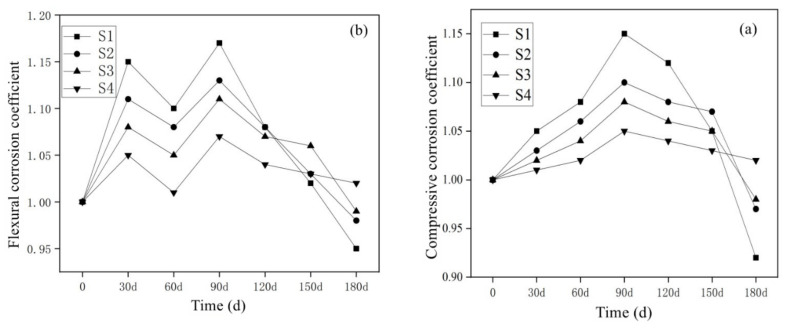
Compressive (**a**) and flexural (**b**) corrosion resistance coefficients of MSWI mortars under sodium sulfate semi-immersion condition.

Figure 9a shows the change of the compressive corrosion resistance coefficient of mortar containing MSWI under long-term semi-immersion in sodium sulfate solution. With the extension of erosion age, the compressive corrosion resistance coefficient of the mortar specimens showed a change rule of first increasing and then decreasing. In the early stage of SO_4_^2−^ ion erosion, SO_4_^2−^ ions entered the mortar structure and participated in the reaction, forming sodium sulfate crystals with filling and compacting effect, and the strength of the specimen increased at this time. However, as the erosion age further increased, the accumulation of sodium sulfate crystals caused the specimen to expand and form micro-cracks, which reduced the strength of the specimen. From the beginning of erosion to the age of 180 d, the compressive corrosion resistance coefficients of the four groups of specimens were S1 < S2 < S3 < S4, and the compressive corrosion resistance coefficients of S1, S2, S3 and S4 at 180 d were 0.92, 0.97, 0.98 and 1.02, respectively, indicating that MSWI powder as an active admixture can resist sulfate erosion in mortar.

Figure 9b shows the change of the flexural corrosion resistance coefficient of the mortar containing MSWI under the long-term semi-immersion of sodium sulfate solution. With the prolongation of the erosion age, the flexural corrosion resistance coefficient of the specimens showed a changing law of first increasing and then decreasing. From the overall change trend of the curve, it can be seen that from the beginning of erosion to 180 d, the bending and corrosion resistance coefficients of the four groups of specimens were S1 < S2 < S3 < S4. Under the condition of a constant w/c ratio, the flexural corrosion resistance coefficient of the specimen increased with the increase in MSWI powder content. When eroded to 180 d, the flexural corrosion resistance coefficients of S1, S2, S3 and S4 are 0.95, 0.98, 0.99, and 1.02, respectively, while the flexural corrosion resistance coefficient curve of the S1 experimental group without powder MSWI decreased faster. However, the flexural corrosion resistance coefficient curves of the experimental groups S2, S3 and S4 decreased slowly.

#### 3.3.2. Results of Cl^−^ Corrosion Resistance of Mortar Containing MSWI

**Figure 10:**
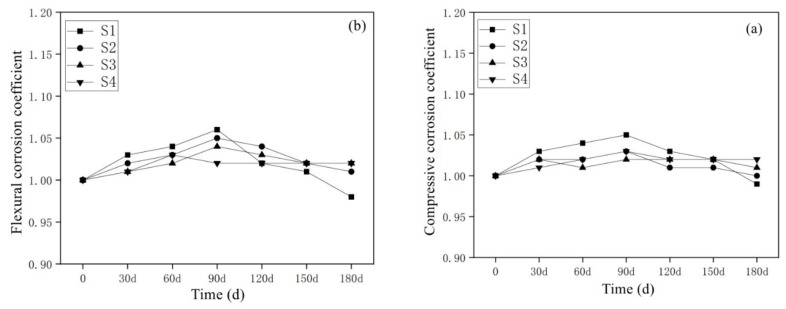
Compressive (**a**) and flexural (**b**) corrosion resistance coefficients of MSWI mortars under sodium chloride semi-immersion condition.

Figure 10 shows the variation law of compressive and flexural corrosion resistance coefficients of MSWI-containing mortar under half-immersion in sodium chloride solution. After long-term immersion in the sodium chloride solution, the compressive and flexural corrosion resistance coefficients of MSWI-containing mortar were all around 1, indicating that Cl^−^ ions did not erode the internal structure of green mortar significantly under semi-immersion conditions. No damage such as spalling and cracking was found on the surface of the MSWI-containing mortar specimens with different contents [35].

**Figure 11:**
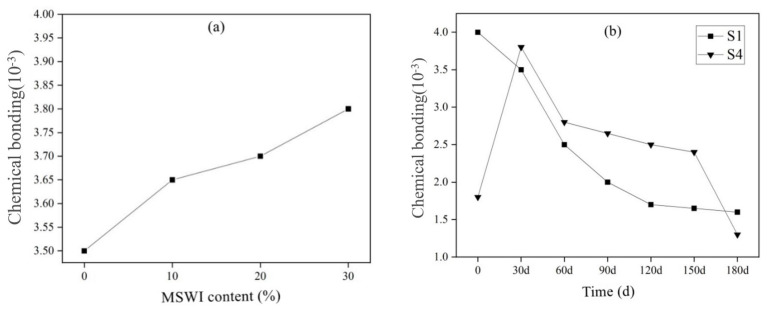
Chloride ion chemical bonding amount of MSWI recycled cement-based material at different dosage (**a**) and age (**b**).

The research showed that the cement hydration products—calcium silicate hydrate (C-S-H) gel—not only had chemical adsorption to chloride ions but also have physical adsorption to chloride ions, and physical adsorption contributes greatly to the total adsorption amount [38]. Since (C-S-H) gel accounts for about 70% of the mass of cement hydration products, the adsorption of chloride ions by hydration products was mainly completed by C-S-H. However, there were few studies on the C-S-H adsorption of chloride ions at present, and the law of C-S-H adsorption of chloride ions was only studied from the composition of cement-based materials [39,40]. In fact, C-S-H was a gel phase with an uncertain calcium-silicon ratio (n (Ca)/n (Si)). When n (Ca)/n (Si) was different, C-S-H had different structures [41]. On the other hand, Zibara et al. [42] found that n (Ca)/n (Si) affected the adsorption of chloride ions by cement hydration products, but the chloride cation promoted the increase in the chain length of calcium silicate hydrate and enhanced its adsorption capacity of chloride ions. Figure 11 shows the chemical binding of chloride ions by cement-based materials with different MSWI powder contents. The chemical binding ability of the mortar with different dosages of MSWI to chloride ions was also different under the standard curing 28 d. The chemical binding amount of S1, S2, S3 and S4 specimen groups was 3.5 mg/g, 3.65 mg/g, 3.7 mg/g, 3.8 mg/g and the chemical binding amount increased with the content of MSWI powder. However, the S4 group mixed with 30% powder showed a trend of first increasing and then decreasing. The chemically bound amount increased from 1.8 mg/g to 3.8 mg/g and then decreased from 3.8 mg/g to 1.3 mg/g.

**Figure 12:**
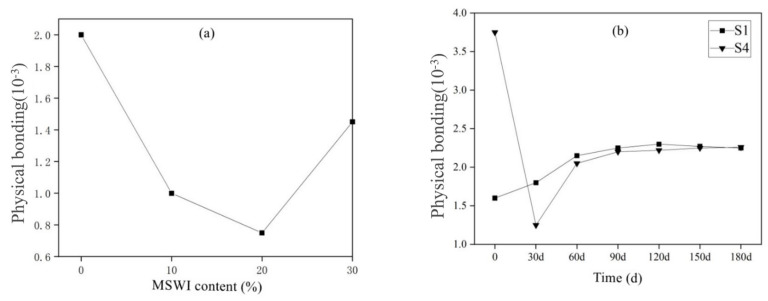
Chloride ion physical bonding amount of MSWI recycled cement-based material at different dosage (**a**) and age (**b**).

Figure 12a shows the variation law of the physical adsorption of chloride ions by cement-based materials with different contents of MSWI. With the increase in MSWI powder content, the physical adsorption capacity of the mortar to chloride ions first decreased from 2 mg/g to 0.75 mg/g. When the dosage was greater than 20%, the physical adsorption capacity increased to 1.45 mg/g. Figure 12b shows the physical adsorption of chloride ions by cement-based materials of different ages. With the increase in age, the physical adsorption of chloride ions by pure mortar in S1 group increased steadily from 1.6 mg/g to 2.25 mg/g. However, the physical adsorption capacity of the mortar samples mixed with 30% powder in the S4 group decreased sharply from 3.75 mg/g to 1.25 mg/g, and the physical adsorption capacity gradually increased after 30 days of age.

**Figure 13:**
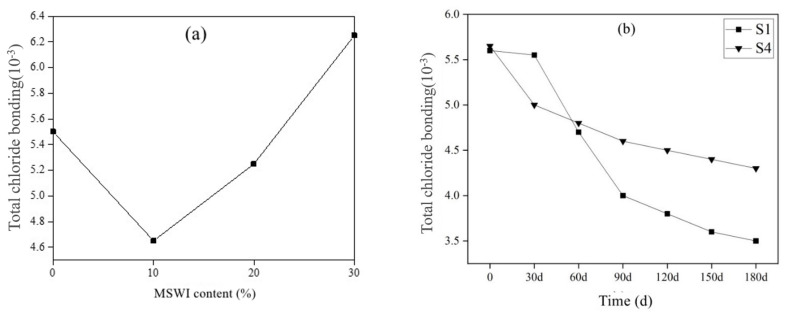
Total chloride ion bonding of MSWI recycled cement-based material at different dosage (**a**) and age (**b**).

Figure 13a shows the total binding amount of chloride ions in cement-based materials with different MSWI powder contents. With the increase in MSWI content, the total combined amount of chloride ions in the mortar gradually increased. The MSWI powder contains active A1_2_O_3_ and SiO_2_, which can undergo hydration reactions with Ca(OH)_2_ in cement-based materials to form hydrated aluminate phase and other derivatives. The aluminate hydrate reacts with chloride ions to form Friede salts, so the cement-based material had a strong chemical combination with chloride ions. Due to the high specific surface area (7000–8000 cm^2^/g) of MSWI powder particles, more chloride ions can be adsorbed on the surface of MSWI particles so that the physical adsorption capacity of MSWI-containing mortar increases.

Figure 13b shows the total combined amount of chloride ions for cement-based materials of different ages. With the increase in curing age, the binding performance of chloride ions of pure cement mortar test block in S1 group decreased with the increase in age. The binding performance of S4 cement-based materials with 30% powder content to chloride ions decreased with the increase in curing age, but the binding capacity of S4 group to chloride ions was still higher than that of the S1 group.

### 3.4. MSWI Recycled Concrete

**Figure 14:**
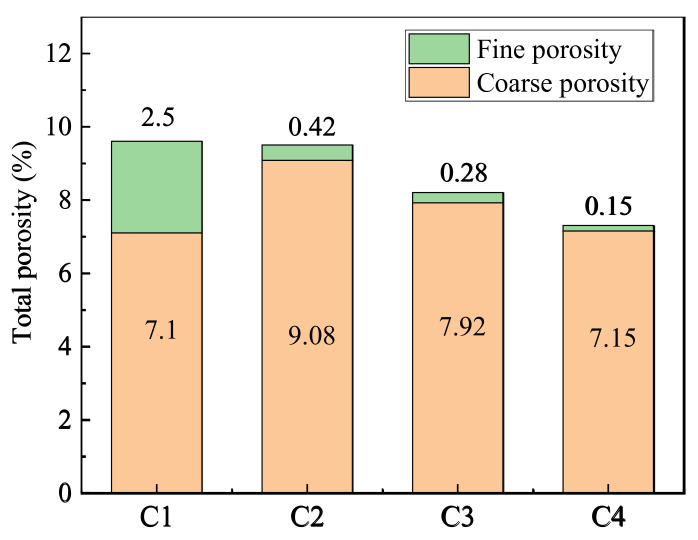
Porosity of recycled concrete at different MSWI content.

The compressive strength test results of concrete specimens containing MSWI are shown in Table 7. With the increase in MSWI powder, the compressive strength of concrete decreased. Figure 14 shows the variation law of porosity of concrete specimens with different MSWI contents. The total capillary porosity, coarse capillary porosity and fine capillary porosity decreased with the increase in powder content. Therefore, the addition of MSWI can effectively reduce the porosity of concrete, especially the coarse capillary porosity.

The pore structure and porosity were key factors affecting the strength of cement-based materials, which were also decisive factors for the resistance of cement-based materials to salt solutions. When the porosity was large and the pores were interconnected, the corrosion resistance of cement-based materials was poor. On the contrary, when the porosity was smaller, the concrete was more compact, and the erosion resistance of concrete can be better obtained. Therefore, the concrete containing MSWI had better corrosion resistance.

**Figure 15:**
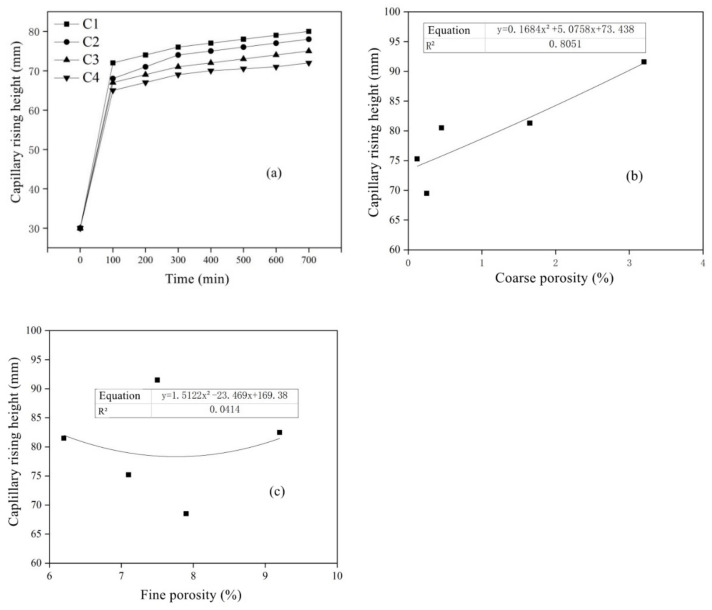
Diagrams of capillary rise height (**a**) versus soaking time, crude porosity (**b**) and system porosity (**c**).

Figure 15a shows the change rule of capillary rise height when concrete containing MSWI is semi-immersed in water. After 700 min, the capillary rise height of pure cement concrete was larger than that of recycled concrete mixed with powder, and the capillary rise height decreased with the increase in MSWI content. Figure 15b,c show the correlation between the capillary rise height and the coarse and fine capillary porosity measured by the evaporable water content method for concrete specimens aged 28 days, with ≥30 nm holes) possessing a good correlation with a correlation coefficient of 0.8051. However, the height of capillary rise had little correlation with fine capillary porosity (pores with pore size < 30 nm and above), with a correlation coefficient of only 0.0414. Therefore, capillary pore types with different pore sizes had different effects on the capillary rise height of concrete, and coarse capillary pores played a key role in the capillary rise transport of solutions in concrete [43].

**Figure 16:**
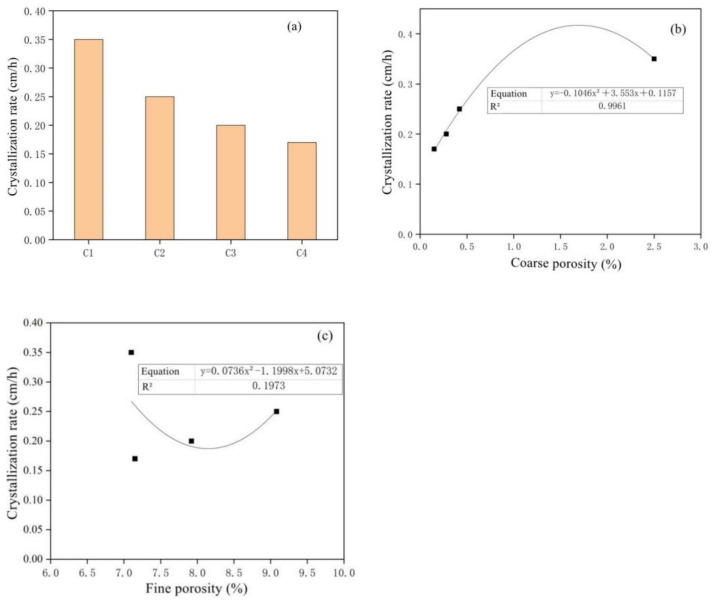
Diagrams of crystallization rate versus MSWI content (**a**), coarse porosity (**b**) and fine porosity (**c**).

Figure 16a shows the change rule of capillary transport crystallization rate of MSWI-containing concrete. The capillary crystallization rate of recycled concrete with different dosages was different. With the increase in powder dosage, the capillary transport rate of recycled concrete gradually decreased. Figure 16b,c show the correlation between the crystallization rate of capillary transport and the coarse and fine capillary porosity. With the increase in powder content, the crystallization rate of capillary transport decreased. The time for the sodium sulfate solution to reach the concrete surface was shorter, resulting in a larger crystalline area on the concrete surface. There was a good correlation between the capillary transport crystallization rate and the coarse capillary porosity, and the correlation coefficient was 0.994. However, the correlation between crystallization rate and fine capillary porosity is not significant, and the correlation coefficient was only 0.197. This explained the importance of coarse capillary porosity in solution capillary transports in concrete.

**Figure 17:**
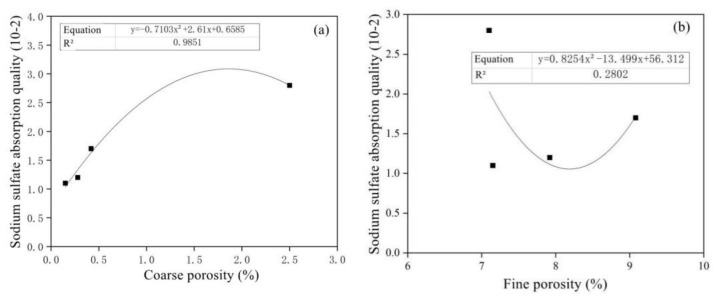
Diagrams of sodium sulfate absorption quality versus coarse (**a**) and fine porosity (**b**).

Figure 17 shows the correlation between the absorbed sodium sulfate solution mass and the coarse and fine capillary porosity. With the increase in powder content, the absorption of sodium sulfate solution in concrete gradually decreased, the absorption of sodium sulfate solution had a good correlation with the coarse capillary porosity, and the correlation coefficient was 0.9851. However, the correlation between the absorption of sodium sulfate solution and the fine capillary porosity is poor, with a correlation coefficient of only 0.280.

## 4. Conclusions

The main conclusions of this paper are as follows:

1. With the increase in MSWI powder content, the compressive strength and flexural strength of mortar specimens at various ages showed a decreasing trend, and the reduction in flexural strength was larger than that of compressive strength. The micro-powder had little influence on the strength of recycled mortar in the early stage, but it had significant influences on the strength in the later stage. The strength activity index of regenerated powder was about 55% when the content of regenerated powder was 30%.
2. With the passage of the sample’s half-soaking time in sodium sulfate solutions, the mass loss rate of mortar decreased gradually with the increase in MSWI content, while the corrosion resistance coefficient increased first and then decreased. Under the condition of constant w/c ratio, the salt crystal erosion resistance of mortar can be improved effectively with the addition of MSWI powder.
3. The cement-based materials containing MSWI had a high ability to bind chloride ions. With the aging of curing, the performance of cement-based materials to bind chloride ions decreased. The total porosity, coarse capillary porosity and fine capillary porosity of recycled concrete decreased with increasing MSWI powder content.
4. The amount of MSWI powder can affect the capillary height, absorption and crystallization rate of sulfate solution in concrete. The capillary rise height, osmotic crystallization rate and 5% sodium sulfate solution were linearly correlated with capillary porosity, but they were linearly independent of capillary porosity; thus, capillary porosity played a key role in capillary transport, migration and the crystallization of sulfate solution in recycled concrete.
5. The MSWI powder had a certain strength activity, and it was feasible to replace part of the cement as a mineral admixture. It was not only beneficial to reduce the environmental problems caused by MSWI bottom slag, in line with the concept of green and sustainable development, but it also can make concrete more dense and improve the SO_4_^2−^/Cl^−^ erosion resistance of concrete.

## Figures and Tables

**Table 1:** Chemical composition of MSWI powder.

Composition	SiO_2_	CaO	Al_2_O_3_	Na_2_O	Fe_2_O_3_	SO_3_	K_2_O	MgO	TiO_2_
Content (%)	48.41	14.78	11.99	3.25	5.40	1.86	1.42	1.78	0.76

**Table 2:** Mix proportion of MSWI mortar.

MSWI (%)	Cement (kg/m^3^)	MSWI (kg/m^3^)	Sand (kg/m^3^)	Water (kg/m^3^)
0	450	0	1350	225
10	405	45	1350	225
12.5	393.75	56.25	1350	225
15	382.5	67.5	1350	225
17.5	371.25	78.75	1350	225
20	360	90	1350	225
22.5	348.75	101.25	1350	225
25	337.5	112.5	1350	225
27.5	326.75	123.75	1350	225
30	315	135	1350	225

**Table 3:** Mix proportion of chloride ion erosion tests of MSWI mortar.

Number	MSWI (%)	Cement (kg/m^3^)	MSWI (kg/m^3^)	Sand (kg/m^3^)	Water (kg/m^3^)
S1	0	450	0	1350	225
S2	10	405	45	1350	225
S3	20	360	90	1350	225
S4	30	315	135	1350	225

**Table 4:** Mix proportion of MSWI concrete.

Number	MSWI (%)	Cement (kg/m^3^)	MSWI (kg/m^3^)	Water (kg/m^3^)	Sand (kg/m^3^)	Gravel (kg/m^3^)	Super-Plasticizer (kg/m^3^)
C1	0	330	0	130	614	1248	2.65
C2	10	297	33	130	614	1248	2.65
C3	20	264	66	130	614	1248	2.65
C4	30	231	99	130	614	1248	2.65

**Table 5:** Compressive strength of MSWI-recycled sand.

MSWI Content (%)	Compressive Strength (MPa)				Strength Activity Index (%)
	3 d	7 d	14 d	28 d	
0	29.37	43.65	53.35	57.61	100
10.0	23.31	34.64	42.17	45.52	79.03
12.5	22.76	34.35	41.92	43.89	76.22
15.0	19.36	31.59	40.60	42.19	73.25
17.5	19.14	28.02	40.57	41.33	73.51
20.0	18.57	27.41	34.62	36.35	62.99
22.5	18.03	27.05	34.26	35.67	61.92
25.0	17.45	26.13	34.20	35.28	61.28
27.0	15.15	23.90	30.96	32.90	57.12
30	15.04	22.83	30.20	32.15	55.80

**Table 6:** Flexural strength of MSWI recycled mortar.

MSWI Content (%)	Flexural Strength (MPa)	Strength Activity Index (%)
10	5.21	58
12.5	5.13	57
15	5.00	55.5
17.5	5.02	55.8
20	4.98	55.3
22.5	4.91	54.6
25	4.82	53.4
27.5	4.62	51.6
30	4.49	49.8

**Table 7:** Compressive strength of MSWI recycled concrete.

Number	W/B Ratio	MSWI Content (%)	Compressive Strength (MPa)
C1	0.4	0	39.5
C2	0.4	10	33.7
C3	0.4	20	27.4
C4	0.4	30	23.9

## Data Availability

Data sharing is not applicable.
